# Long noncoding RNAs, microRNAs, and circular RNAs landscape in hypertensive disorders of pregnancy: progress, challenges, and perspectives 

**DOI:** 10.3389/fgene.2026.1796320

**Published:** 2026-04-28

**Authors:** Ting Xu, Xi Yu, Bingyu Ji, Yueming Zhang, Ruiqing Tong, Qinqin Gao

**Affiliations:** 1 Department of Gynecology and Obstetrics, The Fourth Affiliated Hospital of Soochow University, Suzhou, Jiangsu, China; 2 Institute for Fetology, First Affiliated Hospital of Soochow University, Suzhou, China

**Keywords:** hypertensive disorders of pregnancy, noncoding RNAs, treatment, diagnosis, prediction

## Abstract

Hypertensive disorders of pregnancy (HDP) are a major public health problem that increase morbidity and mortality in many mothers and newborn infants and are considered a significant clinical and economic burden worldwide. The precise pathogenesis of HDP is not yet fully understood, but as the disease progresses, noncoding RNAs (ncRNAs) such as long noncoding RNAs (lncRNAs), microRNAs (miRNAs), and circular RNAs (circRNAs) play abnormal regulatory roles. These ncRNAs function in complex regulatory networks and regulate key biological processes such as cell proliferation, invasion, migration, and apoptosis. Special attention is given to their molecular targets, associated signaling pathways, and the underlying mechanisms of regulatory imbalances. By integrating recent findings and identifying gaps in the main knowledge, this article aims to provide valuable insights into ncRNAs in HDP and to guide therapeutic strategies for this maternal-fetal condition. This will help to establish a profile in this important area of knowledge concerning the clinical management of pregnant women and encourage further research.

## 1 Introduction

Hypertensive disorders of pregnancy (HDP) are one of the leading causes of death among pregnant women and newborn infants, and they are a serious challenge in obstetrics that have profound effects on maternal and infant health. The clinical definition of high blood pressure in pregnancy is a systolic blood pressure ≥140 mmHg and/or a diastolic blood pressure ≥90 mmHg. According to the American College of Obstetricians and Gynecologists (ACOG) classification, HDP are divided into four main categories: chronic hypertension, gestational hypertension, pre-eclampsia/eclampsia (PE), and pre-eclampsia superimposed on chronic hypertension (Hypertension in pregnancy, 2013; Garovic et al., 2022). Among these conditions, PE and preeclampsia superimposed on chronic hypertension are particularly closely linked to an increased clinical risk (Hypertension in pregnancy, 2013), which explains why they have been the subject of extensive research. Around 10%–15% of pregnancies are affected by complications related to high blood pressure. It is the leading cause of high morbidity and mortality among mothers and newborns in low- and middle-income countries (Jiang et al., 2022). HDP was previously regarded as a temporary condition confined solely to gestation. However, PE does not only affect maternal short-term health; it also significantly increases the risk of long-term cardiovascular disease—including coronary heart disease, heart failure, and other cardiovascular disorders (Garovic et al., 2020). Furthermore, HDP has a significant impact on a fetus’s growth and development, as well as their long-term health (Huang et al., 2022; Dines et al., 2023). As high blood pressure affects both the mother’s and the child’s health, elucidating the underlying mechanisms of hypertension and its associated adverse outcomes has become a crucial area of research in maternal and child health; without a full understanding of the disease’s natural history, effective preventative strategies cannot be developed. And it is not possible to develop targeted preventative strategies aimed at reducing its severe short- and long-term complications without a deeper understanding of the pathogenesis of HDP.

Noncoding RNAs (ncRNAs) are a type of functional RNA molecule that does not translate into a protein but regulates gene expression. They play important roles in many biological processes. According to their length, ncRNAs are initially divided into three categories: long noncoding RNAs (lncRNAs, over 200 nucleotides), microRNAs (miRNAs, 20-24 nucleotides), and circular RNAs (circRNAs) (Cech and Steitz, 2014; Nagano and Fraser, 2011). These regulatory molecules orchestrate gene expression through various mechanisms such as transcriptional regulation, splicing and translation control, while simultaneously maintaining genomic structure and stability (Cech and Steitz, 2014; Nagano and Fraser, 2011). Extensive research has shown tha ncRNAs play a central role in fundamental biological processes throughout the life cycle, from ontogeny and reproduction to apoptosis and cellular reprogramming (Beermann et al., 2016; Ali et al., 2021). The importance of ncRNAs regulation in development is clear, and they are promising prognostic markers and therapeutic targets; furthermore, numerous therapeutic strategies targeting ncRNAs are currently being developed for various diseases (Beermann et al., 2016; Ali et al., 2021).

In the context of pregnancy-related diseases, there is growing evidence of a significant link between abnormal regulation of ncRNA expression and HDP, particularly PE (Enquobahrie et al., 2011; Brodowski et al., 2021). Clarifying the expression patterns of ncRNAs in HDP not only provides important insights into the molecular mechanisms of this condition but also opens up possibilities for the development of targeted therapeutic strategies. In this review, we summarize the recent advances in ncRNA research and discuss the intricate interactions in the pathogenesis of HDP, aiming to lay a foundation for future mechanistic studies and clinical applications.

## 2 lncRNAs in hypertensive disorders of pregnancy

In the organism’s genome, noncoding regions are rich in regulatory and functional elements, making an indispensable contribution to the diversity of organisms. Genomic studies have shown that lncRNAs represent a major class of functional noncoding transcripts; their significance is increasingly underscored (Uszczynska-Ratajczak et al., 2018; Derrien et al., 2012). According to the definition, lncRNAs are RNA molecules longer than 200 nucleotides that cannot encode protein (Statello et al., 2021), constituting a highly heterogeneous group of regulatory molecules with diverse structural and functional characteristics. Growing evidence suggests that lncRNAs play a significant role in the regulation of numerous human diseases, including, but not limited to, malignant disorders and cardiovascular diseases (Chi et al., 2019; Uchida and Dimmeler, 2015). With advances in gene expression analysis technology, an increasing number of lncRNAs are being identified as potential participants in the development of HDP, revealing new roles in this complex disease.

HDP is a complex condition with multiple causes, and its exact cause is not well understood. In a normal pregnancy, the early placenta undergoes extensive spiral artery remodeling, a process primarily dependent on the migration and invasion of extravillous trophoblasts (EVTs) and their capacity to replace uterine myometrial endothelial cells and vascular smooth muscle layers (Knöfler et al., 2019). Although this precise process can be easily disrupted, as the pregnancy itself is a very complex and sensitive physiological progression. Studies have shown that changes in trophoblast function, spiral artery remodeling, and oxidative stress are closely associated with high-risk pregnancy (Lyall et al., 2013; Brosens et al., 2019; Rana et al., 2019). In recent years, lncRNAs have been recognized as playing an important role in trophoblast function, spiral artery remodeling, and oxidative stress, offering new insights into the fundamental molecular mechanisms underlying the development of HDP.

### 2.1 The role of lncRNAs in modulating trophoblast cell functions

**FIGURE 1:**
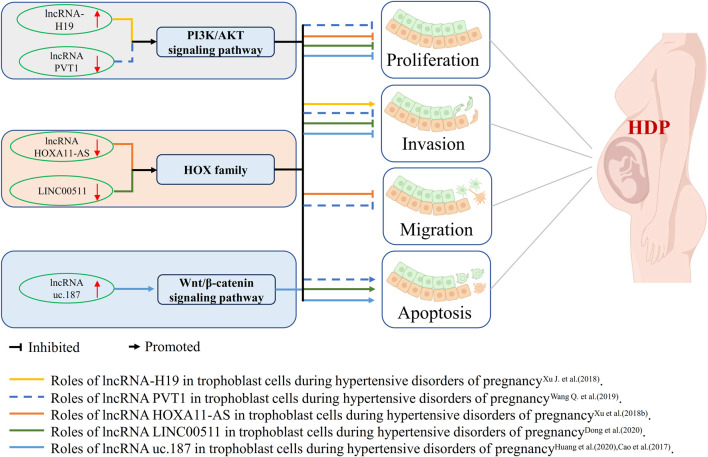
lncRNA Dysregulation and Roles in Trophoblast Cells During HDP.

Normal placental function is essential for a successful pregnancy and to support proper fetal development in the uterus. Dysfunction of placentation poses a serious risk to the health of the mother and fetus, causing pregnancy-related complications, including PE (Xiao et al., 2020; Fisher, 2015). The function of trophoblast cells, the main cell type driving the development of the placenta, is crucial for maintaining the physiological balance and function of the placenta. Recent studies have shown that dysregulation of ncRNAs is closely linked to changes in the behavior and function of trophoblast cells, which is mediated through three primary signaling pathways: the PI3K/AKT/mTOR axis, pathways associated with the HOX family, and the Wnt/β-catenin pathway. A brief overview of these findings is presented in Figure 1, and they are discussed in detail in the following sections.

Trophoblast cells perform several functions, such as proliferation, invasion, migration, and apoptosis, which are essential for the healthy development of the placenta. Dysfunctions in one or more of these cellular processes are often observed in diseases. Further research has shown that lncRNAs can regulate the behavior of trophoblast cells via the PI3K/AKT/mTOR signaling pathway, a key regulator of cellular function. For example, Xu et al. demonstrated that lncRNA-H19 in the placentas of patients with PE is significantly upregulated and helps to regulate the proliferation of trophoblast cells. Their results showed that JEG-3 and HTR-8 trophoblast cell lines with high expression of lncRNA-H19 exhibited reduced cell viability and increased apoptosis and autophagy (Xu J. et al., 2018). However, Wang and colleagues have reported that the lower expression of lncRNA PVT1 in women with PE leads to regulation of the PI3K/AKT pathway, causing trophoblast cell apoptosis and also reducing their proliferation, invasion, and migration (Wang Q. et al., 2019). Although there are some differences in these conclusions—in particular, another group of researchers found reduced activity of lncRNA PVT1 in severe cases of PE—their study further clarified that an active interaction between lncRNA PVT1 and the epigenetic repressor Ezh2, which limits ANGPTL4 expression and, consequently, impairs trophoblast cell function (Xu et al., 2018a).

The HOX transcription factor family, a distinct subgroup within the homeobox superfamily, plays a crucial role in embryonic morphogenesis and the maintenance of adult tissue homeostasis (Yu et al., 2020). Extensive research shows that the HOX family members are closely involved in the processes of cellular proliferation and metastasis and that their overexpression is evident in many types of cancer (Shah and Sukumar, 2010). Notably, recent studies have discovered abnormal expression of the HOX family members in the placental tissue of patients with PE. This indicates that they may be involved in the dysregulation of trophoblast function. A marked reduction in the expression of the lncRNA HOXA11-AS has been observed in the placentas of patients with PE, and functional studies have shown that HOXA11-AS regulates the expression of RND3 and HOXA7, which alter the proliferation and migration of trophoblast cells (Xu et al., 2018b). In addition, Dong and his team reported that HOXA7 expression was reduced in the placental tissues with PE and that this was positively correlated with LINC00511 expression. Their mechanistic studies showed that downregulation of LINC00511 reduces trophoblast proliferation, autophagy, and invasion but increases apoptosis (Dong et al., 2020).

The Wnt/β-catenin signaling pathway is essential in maintaining adult tissue homeostasis and embryonic development. An imbalance in this pathway leads to the development of numerous diseases (Liu J. et al., 2022). Huang et al. showed that increased expression of the lncRNA uc.187 during pregnancy promotes the onset of PE in rats. Their study also shows that the abnormal biological behavior of HTR-8/SVneo trophoblast cells is associated with altered subcellular distribution of β-catenin, which is characterized by abnormal accumulation in both the cytoplasm and the nuclear compartments (Huang et al., 2020). These findings are consistent with previous reports showing the overexpression of lncRNA uc.187 in the placental tissues of patients with PE (Cao et al., 2017). Moreover, Cao and colleagues provided evidence that in HTR-8/SVneo cells, silencing lncRNA uc.187 obviously upregulated the expression of proteins associated with invasion and proliferation, whilst inhibiting apoptosis-related proteins (Cao et al., 2017). These studies colud demonstrate that lncRNA uc.187 plays a crucial role in the pathogenesis of PE by regulating the Wnt/β-catenin signaling pathway.

In addition to regulating trophoblast cellular functions through the above canonical molecular pathways, a specific subset of lncRNAs exacerbates the progression of HDP via other signaling axes. According to the timing of delivery, PE can be classified into two types: early-onset PE (EOPE) means a delivery before 34 weeks of pregnancy, while late-onset PE (LOPE) refers to a delivery at or after 34 weeks of pregnancy. EOPE significantly increases the risk of morbidity and mortality for both mother and child in the short and long term (Poon et al., 2019). Recent studies have shown a close association between the aberrant lncRNA expression in the EOPE placenta and impaired trophoblast function. As Song reported, lncRNA uc.294 is significantly upregulated in EOPE placental tissues, where it suppresses trophoblast cell proliferation and invasion while promoting apoptosis (Song et al., 2019). Furthermore, Qin et al. showed that the downregulation of lncRNA MALAT1 in EOPE controls the migration and invasion of trophoblast cells by modulating the FOS gene (Li et al., 2020). However, current studies have focused most on lncRNA-mediated regulation of trophoblast function, with merely their key target genes identified thus far. Further mechanistic studies are therefore required to clarify the specific downstream signaling pathways.

If the abnormal expression of lncRNAs dysregulated the function of trophoblasts, which is the primary trigger for the onset of HDP; then abnormal remodeling of the spiral arteries is the main pathophysiological process that leads to placental ischemia. This key concept will be further explained in the next section.

### 2.2 The emerging role of lncRNAs in uterine spiral artery remodeling

It is well known that the failure of uterine spiral artery remodeling, leading to placental ischemia, is the main pathophysiological process in PE. This vascular remodeling defect is associated with abnormal communication among trophoblast cells, vascular endothelial cells, and vascular smooth muscle cells (VSMCs). The defection of extravillous trophoblastic invasion and abnormal remodeling of spiral arteries are key contributors in HDP (Lyall et al., 2013; Xiao et al., 2020). The lncRNAs have emerged as critical upstream regulators of this multicellular communication network, governing the entire process of spiral artery remodeling via conserved regulatory mechanisms rather than isolated molecular events. Cumulative evidence confirms that dysregulated lncRNAs uniformly disrupt spiral artery remodeling by targeting core trophoblast behaviors, including proliferation, migration, invasion, and apoptosis, with most functioning as competitive endogenous RNAs (ceRNAs) to sequester target miRNAs. Some lncRNAs, such as lncRNA ATB, NR_002794, and ZBTB39-1:2, show dysregulation in the expression of placental tissues in PE, and their dysregulation directly affects trophoblast invasion capacity and/or alters endothelial cell coordination, ultimately impeding normal vascular vessel remodelling (Yin et al., 2021; Ma et al., 2019; Liu Y. et al., 2018).

Except for these regulatory molecules, lncRNA TUG1 stands out as a pleiotropic regulator, impairing spiral artery remodeling through multiple parallel pathways: mediated by the miR-218/FOXP1 axis, epigenetically suppressing RND3 to modulate trophoblast activity, and regulating endothelial function via the miR-29a-3p/VEGFA and Ang2/Tie2 signaling axes (Xu et al., 2022; Xu et al., 2017; Liu Z. et al., 2022). The impact of lncRNAs, however, extends beyond trophoblasts to directly affect vascular cells. For example, low expression of lncRNA HIF1A-AS2 via the HIF1A-AS2–Lamin A/C–ANGPTL4 axis exacerbated the deterioration in vascular remodelling by inhibiting proliferation, migration, and tubule formation of trophoblasts and vascular endothelial cells (Shu L. et al., 2022).

The migration of EVTs drives vascular smooth muscle cell depletion, serving as a critical mechanism for successful uterine spiral artery remodeling during placental development (Robson et al., 2019). In this context, the tumor suppressor lncRNA MEG3 has been shown to play a pivotal role, with its expression drastically reduced (up to 80% downregulation) in PE placental tissues (Zhang et al., 2015). Liu et al. demonstrated that lncRNA MEG3 is regulated by uterine natural killer cell-derived TGF-β1 to control VSMC migration and apoptosis (Liu W. et al., 2019). A subsequent independent validation confirmed these findings (Liu et al., 2017). MEG3 dysregulation triggers a cascade of pathological effects, promoting apoptosis in VSMCs, trophoblasts, and HUVECs; activating the NF-κB, Caspase-3, and Bax apoptotic signaling pathways; and inhibiting trophoblast epithelial-mesenchymal transition (EMT) via the miR-210/MEG3 axis, meaning its downregulation coordinately disrupts the function of trophoblasts, endothelial cells, and VSMCs simultaneously (Zhang et al., 2015; Liu W. et al., 2019; Wang et al., 2021).

Collectively, these results provide a regulatory system: lncRNA dysregulation affects the normal spiral artery remodeling and drives the development of PE by destabilizing the function of multiple cell types, as shown in Table 1. However, the disruption of vascular remodeling is only one facet of lncRNA-mediated pathophysiology in HDP.

**TABLE 1:** Expression Profiles of lncRNAs Implicated in Inhibition of Uterine Spiral Artery Remodeling.

lncRNAs	Expression levels in HDP	Detail mechanism	Function of the trophoblast cells	Ref.
lncRNA ATB	Down	lncRNA ATB↓→miR-651–3p↑→YY1↓	Inhibited invasion and migration (trophoblast cells)	Yin et al. (2021)
lncRNA NR_002794	Up	—	Inhibited proliferation and migration, but promoted apoptosis (trophoblast cells)	Ma et al. (2019)
lncRNA ZBTB39-1:2	Up	—	Inhibited migration (trophoblast cells)	Liu Y. et al. (2018)
lncRNA TUG1	Down	miR-218↑→FOXP1↓→lncRNA TUG1↓	Inhibited migration (trophoblast cells)	Xu et al. (2022)
	Down	lncRNA TUG1→Ezh2→RND3↑	Inhibited proliferation, invasion and migration, but promoted apoptosis (trophoblast cells)	Xu et al. (2017)
	Down	lncRNA TUG1↓→miR-29a-3p↑→VEGFA and Ang2-Tie2↓	Inhibited proliferation, invasion, migration, and angiogenesis (HUVECs)	Liu Z. et al. (2022)
lncRNA HIF1A-AS2	Down	FOXP1↓→lncRNA HIF1A-AS2↓→Lamin A/C↓→ANGPTL4↑	inhibited cell proliferation, migration, invasion and angiogenesis (trophoblast cells and HUVECs)	Shu L. et al. (2022)
lncRNA MEG3	Down	—	Inhibited migration, but promoted apoptosis (trophoblast cells and HUVECs)	Zhang et al. (2015)
	Down	uNK‐derived TGF‐β1↓→lncRNA MEG3↓	promoted proliferation, but inhibited apoptosis and migration (VSMCs)	Liu W. et al. (2019)
	Down	dNK‐derived IFN-γ↓→lncRNA MEG3↓	promoted proliferation, but inhibited migration (VSMCs)	Liu et al. (2017)
	Down	miR-210↑→lncRNA MEG3↓	Inhibited migration and invasion (trophoblast cells)	Wang et al. (2021)

### 2.3 Dysregulated lncRNAs drive oxidative stress in HDP pathogenesis

In addition to its role in the remodeling of the uterine spiral artery, lncRNA also functions as another key pathogenic factor in HDP: oxidative stress. The intricate interplay between oxidative stress and HDP has been recognized as a primary cause of cardiovascular disease, and there is considerable evidence highlighting the essential role of oxidative stress in HDP (Brosens et al., 2019; Rana et al., 2019). Emerging research has established that lncRNAs act as key mediators in this process, orchestrating redox homeostasis and oxidative stress-related inflammatory responses in the placental microenvironment, with consistent regulatory patterns emerging across independent studies despite minor phenotypic discrepancies. The lncRNAs disrupt cellular antioxidant capacity by targeting key redox and inflammatory genes via ceRNA regulatory networks. The lncRNA MALAT1, one of the most extensively studied proteins in this field, is associated with oxidative stress and inflammation; it acts as a ceRNA for miR-150–5p, which upregulates the expression of endothelin-1, finally leading to oxidative imbalance and accelerating the progression of HDP (Ou M. et al., 2020). Notably, inconsistent serum MALAT1 expression levels have been reported in PE patients (Li et al., 2020; Abdelazim et al., 2022), with one study documenting reduced expression correlating with disease severity (Abdelazim et al., 2022). This discrepancy underscores several major limitations in current lncRNA research. First, there were marked differences of clinical ethnicity and sample size among HDP patient cohorts, (e.g., the former study enrolled 55 Chinese participants, while the latter included 82 Egyptian individuals). Second, the two studies differed in their inclusion criteria. For instance, the former collected maternal blood samples at 32–40 gestational weeks, whereas the latter obtained samples from 20 to 40 gestational weeks and also recruited women with severe complications; such details were not explicitly reported in the former study. Third, species variability between human and rat model samples, which is a common source of conflicting results in ncRNA studies (mechanistic investigations in the former study were primarily conducted in animal models). Those discrepancies highlighting the need for large-scale, multicenter cohort studies with standardized patient stratification criteria to resolve these inconsistencies.

Beyond MALAT1, other lncRNAs have also been shown to regulate oxidative stress in HDP. For example, it has been shown that LINC00240 levels are consistently reduced in the placental tissues of patients with PE and in experimental models. Functional analysis showed that the LINC00240/miR-155/Nrf2 axis triggers oxidative stress-mediated pyroptosis, which affects trophoblast proliferation and invasion (Wu et al., 2022). Although these results are significant, the current studies are still limited to identifying individual molecular axis characterization, and no large-scale studies have been conducted to investigate the relationship between global redox regulatory networks and various lncRNAs in response to oxidative stress. This gap is further deepened by limited validation studies in large cohorts, hindering clinical application.

### 2.4 Possibility: lncRNAs as potential targets for HDP

According to the aforesaid research, which has illuminated the critical involvement of lncRNAs in the pathogenesis of HDP, particularly through their regulatory roles in trophoblast cell proliferation, invasion, migration, apoptosis, spiral artery remodeling, and oxidative stress responses. Given the substantial morbidity associated with HDP, the identification of reliable biomarkers for early prediction and therapeutic intervention represents a pressing clinical imperative.

The present research has primarily focused on the comparative analysis of the expression of lncRNAs from patients with HDP and control groups; evidence confirmed the potential of lncRNAs to become the therapeutic targets. Circulating lncRNAs in peripheral blood are extremely promising as non-invasive biomarkers, owing to their intrinsic stability and the ease of sample collection (Dai et al., 2021; Wu and Su, 2022; Sun et al., 2019).

However, several important limitations hinder their clinical application: Firstly, small sample sizes, single-center studies, and limited validation in diverse population groups. Tissue and body fluid biopsy profiles often show inconsistent expression patterns. It is important to conduct large-scale, multi-center validation studies and to develop standardized protocols for sampling, processing, and quantitative preparation to minimize variability. Secondly, it is necessary that the research shift from a descriptive expression profiling to the complete molecular mechanisms and regulatory networks of HDP-associated lncRNAs, including their interactions with other ncRNAs. Thirdly, conflicting reports in the literature must be resolved through careful comparative analysis, accounting for cohort heterogeneity, sample type variability, and publication bias, avoiding missing out on truly meaningful conclusions. Finally, there are limited preclinical studies investigating targeted therapeutic strategies, and this represents a significant translational gap. Addressing these priorities will not only deepen the understanding of HDP pathogenesis but will also promote non-invasive prenatal diagnostic biomarkers and personalized, targeted treatments—to improve outcomes for the maternal and neonatal through targeted therapies for patients affected by HDP.

## 3 The miRNAs in hypertensive disorders of pregnancy

In addition to lncRNAs, miRNAs represent another major class of noncoding regulators that are strongly implicated in HDP. The miRNAs are noncoding endogenous RNAs in organisms, typically 18 to 25 nucleotides long, that play a key role in the post-transcriptional repression of genes (Liu et al., 2014; Pozniak et al., 2022). MiRNAs, according to the principle of complementary pairing, bind to complementary target sequences in mRNA and then, through affecting the functions of degradation and translational repression of mRNA, regulate target gene expression (Liu et al., 2014; Pozniak et al., 2022). There is growing evidence that abnormal miRNA expression levels are associated with multiple adverse pregnancy outcomes, particularly in HDP. It is also known that some placenta-specific miRNAs are found in the maternal blood, and thus these molecules act as key mediators in the onset of HDP and are also promising noninvasive biomarkers (Luo et al., 2009; Zhou and Xie, 2020). Synthetic analysis shows that miRNAs regulate the development of HDP in three convergent, interconnected pathways: dysregulation of trophoblast cells, damage to the function of the vascular endothelial cell, and a disruption of oxidative stress and angiogenic networks.

### 3.1 The role of miRNAs in modulating trophoblast cell functions

Trophoblast cells are essential for placental function and play an important role in the development of HDP. Numerous studies show that miRNAs regulate trophoblast invasion, apoptosis, migration, and cell proliferation, with nearly all dysregulated miRNAs in HDP converging on these critical phenotypes. Here, based on the difference of trophoblast cell functions, a summary of the miRNAs in HDP is provided in Table 2.

**TABLE 2:** Summary of miRNAs in HDP Categorized by Trophoblast Cell Functions.

Cell type	Function	miRNAs
Trophoblast cells	Invasion	miR-210 miR-34a miR-30a-3p miR-23a miR-200a miR-519d-3p miR-31–5p miR-149–5p miR-218–5p miR-376c miR-576–5p miR-27b-3p
	Apoptosis	miR-34a miR-23a miR-30a-3p miR-204–5p miR-200a miR-195
	Proliferation	miR-384 miR-210–5p miR-204–5p miR-200a miR-576–5p miR-376c
	Migration	miR-384 miR-23a miR-210–5p miR-200a miR-519d-3p miR-218–5p miR-155 miR-376c
Endothelial Cells	—	miR-31–5p miR-27b-3p miR-155 miR-125b-5p miR-150–5p

Specifically, synthetic analysis of independent cohort studies reveals a consistent dichotomy in miRNA-mediated trophoblast regulation: a panel of miRNAs is consistently upregulated in HDP placental and/or serum samples, acting as pathological suppressors of trophoblast motility and survival, while a separate subset is downregulated, losing its physiological promotion of normal functions of trophoblast cells. Collectively, these dysregulated miRNAs target overlapping downstream signaling cascades and effector genes, including apoptotic regulators, cell cycle mediators, and matrix remodeling enzymes, to coordinately impair trophoblast function rather than acting through isolated molecular effects. For example, miR-210, miR-34a, miR-30a-3p, miR-519d-3p, miR-23a, miR-200a, miR-384, miR-210–5p, miR-150–5p, and miR-204–5p are overexpressed in the placental tissue and/or in the maternal serum of HDP patients, where they inhibit the invasion, migration, and proliferation of trophoblast cells; promote apoptosis; or suppress cell cycle progression through various signaling pathways (Ou M. et al., 2020; Anton et al., 2013; Ahn et al., 2017; Guo et al., 2017; Liu J. J. et al., 2019; Niu et al., 2018; Ding et al., 2015; Fan et al., 2020; Wu et al., 2020; Zhou W. et al., 2020; Awamleh and Han, 2020; Mei et al., 2017). It should be noted that a large number of miRNAs have multiple roles, regulating both cell apoptosis and invasion; for example, miR-34a and miR-200a not only inhibit trophoblast cell invasion but also activate pro-apoptotic signaling cascades (Guo et al., 2017; Liu J. J. et al., 2019; Wu et al., 2020). The miR-34a promotes the apoptosis of trophoblast cells by inhibiting the expression of B-cell CLL/lymphoma 2 and inhibits invasion via the Notch signaling pathway (Guo et al., 2017; Liu J. J. et al., 2019). The miR-30a-3p induces trophoblast apoptosis and reduces invasion by downregulating insulin-like growth factor 1 (IGF-1), thus contributing to HDP (Niu et al., 2018).

In addition to regulating trophoblast invasion and apoptosis, the abilities of proliferation and migration are equally vital for the development of HDP, and the misregulation of miRNAs disrupts these key functions and exacerbates placental pathology. For instance, the aberrant upregulation of miR-384 has been consistently linked to altered trophoblast migration via the involvement of polypyrimidine tract-binding protein 3 (PTBP3), which is the key target mediating this inhibitory effect (Zhou W. et al., 2020). In EOPE, upregulation of miR-210–5p directly inhibits the proliferation and migration of HTR-8/SVneo trophoblasts and further aggravates the defects in placental vascularization. (Awamleh and Han, 2020) Additional miRNAs, such as miR-519d-3p and miR-204–5p, regulate trophoblast function by targeting key proteases, including matrix metalloproteinases (MMPs), or the core cell cycle, which is essential for the remodeling of placental blood vessels (Ding et al., 2015; Mei et al., 2017). All the details for each miRNA, its target genes, and expression patterns are summarized in Table 3.

**TABLE 3:** Summary of upregulated miRNAs, Target Genes and Expression Profiles in HDP.

miRNAs	Expression levels in HDP	Sample	Target/Pathway	Function of the trophoblast cells	Ref.
miR-210	Up	serum and placental tissue	ERK/MAPK Pathway	inhibited invasion	Anton et al. (2013)
	Up	trophoblast cells	IGFBP3	—	Ahn et al. (2017)
miR-34a	Up	placental tissue and trophoblast cells	BCL-2	promoted apoptosis	Guo et al. (2017)
	Up	placental tissue and trophoblast cells	Notch-1	inhibited invasion	Liu J. J. et al. (2019)
miR-30a-3p	Up	placental tissue and trophoblast cells	IGF-1	inhibited invasion but promoted apoptosis	Niu et al. (2018)
miR-519d-3p	Up	serum, placental tissue, and trophoblast cells	MMP-2	inhibited invasion and migration	Ding et al. (2015)
miR-23a	Up	placental tissue and trophoblast cells	HDAC2	inhibited invasion and migration, but promoted apoptosis	Fan et al. (2020)
miR-200a	Up	placental tissue and trophoblast cells	ZEB1	inhibited invasion, proliferation, and migration, but promoted apoptosis	Wu et al. (2020)
miR-384	Up	trophoblast cells	PTBP3	inhibited proliferation and migration	Zhou W. et al. (2020)
miR-210-5p	Up	trophoblast cells	CSF1 and ITGAM	inhibited migration	Awamleh and Han (2020)
miR-204-5p	Up	serum and trophoblast cells	—	inhibited proliferation but promoted apoptosis	Mei et al. (2017)
miR-31-5p	Up	serum and HUVECs	eNOS	endothelial cells dysfunction	Kim et al. (2018)
miR-155	Up	serum, placental tissue, HUVECs, and trophoblast cells	eNOS	endothelial cells dysfunction	Kim et al. (2017)
miR-27b-3p	Up	serum, HUVECs, and trophoblast cells	ATP2B1	endothelial cells dysfunction	Zhu and Liu (2021)
miR-150-5p	Up	placental tissue, and trophoblast cells	CYR61	inhibited migration	Zhang et al. (2010)

Conversely, miRNAs such as miR-149–5p, miR-218–5p, miR-576–5p, and miR-376c are downregulated in PE placentas, losing their physiological ability to promote trophoblast invasion and proliferation, thereby exacerbating pathological placental development (Xiaobo et al., 2019; Brkić et al., 2018; Wang et al., 2020; Fu et al., 2013). Of note, Wang et al. reported that the reduced expression of miR-576–5p upregulates transcription factor AP-2α (TFAP2A) and then inhibits trophoblast invasion and proliferation in rat models of PE and trophoblast cells (Wang et al., 2020). More details of individual miRNAs are shown in Table 4.

**TABLE 4:** Summary of downregulated miRNAs, Target Genes and Expression Profiles in HDP.

miRNAs	Expression levels in HDP	Sample	Target/Pathway	Function of the trophoblast cells	Ref.
miR-149-5p	Down	placental tissue	endoglin	inhibited invasion	Xiaobo et al. (2019)
miR-218-5p	Down	placental tissue	TGF-β2	inhibited invasion	Brkić et al. (2018)
miR-576-5p	Down	PE rats and trophoblast cells	TFAP2A	inhibited proliferation and invasion	Wang et al. (2020)
miR-376c	Down	serum and placental tissue	ALK5 and ALK7	inhibited proliferation and invasion	Fu et al. (2013)
miR-548c-5p	Down	serum and placental tissue	PTPRO	inhibited the proliferation and activation of macrophages	Wang Z. et al. (2019)
miR-125b-5p	Down	HPMECs and rats	BMF	endothelial cells dysfunction	Zheng et al. (2022)
miR-195	Down	serum, placental tissue, and trophoblast cells	FOXRED1 and PDPR	promoted apoptosis	Wang et al. (2018)

Whether these mechanistic findings apply to HDP of human pregnancy and have clinical relevance remains to be confirmed through large-scale studies and primary tissue validation. Because a large proportion of supporting data is derived from immortalized trophoblast cell lines and rodent models of PE, both of which fail to fully recapitulate the physiological complexity of human placentation. Immortalized trophoblast cell lines fail to fully reproduce the invasive phenotype of primary human EVTs derived from first-trimester or full-term placentas and thus could cause potential overestimation or misinterpretation of miRNA (Abbas et al., 2020). Moreover, the anatomical and physiological differences between rodent and human placentation are profound: rodents possess a hemotrichorionic placenta with distinct structural organization, vascular remodeling dynamics, and trophoblast lineage differentiation pathways compared to humans. Therefore, mechanistic inferences drawn from rodent models cannot be directly applied to human pregnancy without profound validation (Abbas et al., 2020; Serman and Serman, 2011). Despite the limitations of animal models and immortalized trophoblast cell lines, it does not mean that they should be entirely discarded. Among existing research methods, they still offer distinct advantages in studying placental dysfunction. Accordingly, it is imperative to dissect the pathological contributions of ncRNAs to HDP onset and progression using diversified, multi-level research methodologies and to avoid biased or irreproducible findings stemming from single-model research.

Overall, these results indicate that miRNAs exert a multifaceted effect on trophoblast function via various molecular pathways and play a significant role in the pathogenesis of HDP. However, except for trophoblast cell dysfunction, their effects on endothelial cells are also no less important in the pathophysiology of HDP.

### 3.2 The role of miRNAs in modulating endothelial cell functions

Beyond trophoblasts, miRNAs also govern maternal vascular function, and endothelial dysfunction is a hallmark of HDP. Endothelial dysfunction is defined as a pathogenic imbalance between proinflammatory cytokines and decreased anti-inflammatory cytokines, which plays an important role in the pathogenesis of HDP (Sakowicz et al., 2023; McElwain et al., 2020). Emerging evidence highlights the regulatory roles of miRNAs in regulating the functions of endothelial cells, as shown in Table 3. Synthetic analysis of existing research unifies disparate findings into a core mechanistic theme: the majority of HDP-associated miRNAs promote endothelial dysfunction by enhancing pro-inflammatory cascades. The TNF-mediated NF-κB signaling, usually activated by pro-inflammatory miRNAs, then triggers the release of pro-inflammatory cytokines, finally perpetuating systemic vascular inflammation (Kim et al., 2018; Wang Z. et al., 2019). Such as, Suji Kim and colleagues found that serum miR-31–5p concentrations were 2.7 times higher in patients with PE. This miRNA accelerates the progression of PE by activating the NF-κB pathway through the upregulation of the proinflammatory cytokine TNF-α (Kim et al., 2018). Besides, aspirin could attenuate NF-κB-dependent miR-155 host gene (MIR155HG) expression, ameliorating endothelial dysfunction in PE (Kim et al., 2017). Intriguingly, experimental data show that the downregulation of miR-548c-5p disrupted the balance between inflammatory factors by targeting PTPRO and led to the occurrence of PE, indicating that it may exert anti-inflammatory effects in the progression of PE (Wang Z. et al., 2019).

Trophoblast dysfunction and endothelial impairment are not independent pathological events in HDP, increased expression of miR-27b-3p in PE not only increases trophoblastic cell invasion but also promotes proliferation, migration, and tube formation of vascular endothelial cells by suppressing the expression of ATPase plasma membrane Ca^2+^ transporting 1 (ATP2B1) (Zhu and Liu, 2021). And the miR-125b-5p overexpression downregulated the BMF gene, reduced hypertension symptoms, and improved pregnancy outcomes by affecting the function of vascular endothelial cells (Zheng et al., 2022). These findings highlight that miRNAs modulate both inflammatory and angiogenic activities in endothelial cells, thereby disrupting the normal endothelial adaptation to pregnancy, perpetuating placental ischemia and amplifying systemic disease severity.

### 3.3 Interplay between miRNAs and other molecular pathways in HDP

The miRNAs do not function in isolation within the HDP pathological microenvironment; instead, they act as central regulatory factors within complex networks that interaction with oxidative stress, angiogenesis, and lncRNA-mediated signaling. In the development of HDP, oxidative stress is initiated with dysregulated miRNAs disrupting mitochondrial function and cellular redox homeostasis to induce trophoblast apoptosis, placental injury, and vascular damage, such as miR-195, miR-150–5p, and miRNA-155 (Ou M. et al., 2020; Wang et al., 2018; Zhang et al., 2010). For example, numerous studies have shown that miRNAs accelerate these processes through a mechanism of oxidative stress. In particular, miR-195 can inhibit the flavin adenine dinucleotide (FAD)-dependent oxidoreductase domain-containing protein 1 (FOXRED1) and pyruvate dehydrogenase phosphatase regulatory subunit (PDPR), thereby inhibiting mitochondrial energy synthesis, causing oxidative stress, and leading to trophoblast cell apoptosis (Wang et al., 2018). The miRNAs also operate within complex regulatory networks and affect the redox homeostasis in HDP. The upregulation of lncRNA MALAT1 is competitively bound by miR-150–5p, which downregulates endothelin-1, disrupting redox homeostasis and ultimately leading to HDP (Ou M. et al., 2020).

Among HDP-associated miRNAs with pleiotropic pathological effects, miR-155 has emerged as a key multifunctional regulator. Except for the previously known significant function of miRNA-155 in the inflammatory response (Kim et al., 2017), miR-155 also promotes the pathogenesis of PE by inhibiting angiogenesis. Zhang and colleagues demonstrated that miR-155 inhibits cysteine-rich protein 61 (CYR61), which is a major modulator of angiogenesis, thereby impairing the ability of blood vessels to accommodate patients with PE (Zhang et al., 2010). Clinical observations revealed that elevated miR-155 levels were significantly higher in severe PE patients versus mild cases (Jairajpuri et al., 2017). Mechanistic studies showed that the Forkhead box O3a (FOXO3a), a gene correlated with blood pressure regulation, is inhibited by the upregulated expression of miR-155 (Liu D. F. et al., 2018). These examples illustrate miR-155 is a promising candidate biomarker for future diagnosis strategy development in HDP, pending further rigorous validation in preclinical models and prospective human studies.

Despite these ongoing findings, key limitations remain in the existing literature; most studies focus on specific miRNAs and their pathways, but only a few studies investigate widespread regulatory networks and the collaborative action of multiple ncRNA species.

### 3.4 Possibility: miRNAs as potential targets for HDP

The unique stability and persistence of circulating miRNA molecules have made them reliable biomarkers for early disease diagnosis and monitoring disease progression, offering new insights into patient management. Current research has demonstrated its great importance in regulating gene expression networks and has shown its potential as a novel therapeutic tool within HDP therapy.

Recent studies have extensively explained the role of miRNAs in the development of HDP and shown the comprehensive investigation of the miRNAs in endothelial dysfunction, placental angiogenesis, and systemic inflammation. Specifically, compared to normotensive pregnant women, HDP patients have dysregulated expression levels of miR-155, miR-548c-5p, and miR-31–5p, as well as other miRNAs (Kim et al., 2018; Wang Z. et al., 2019; Jairajpuri et al., 2017). On the other hand, preclinical research offers preliminary proof of concept for therapeutic targeting of miRNA-mediated endothelial dysfunction, with interventions such as aspirin shown to attenuate disease-associated miRNA expression and alleviate vascular inflammation, providing a translational foothold for future strategies (Kim et al., 2017). Taken together, the cumulative findings of these mechanistic studies provide a solid foundation and a compelling rationale for conducting more in-depth research in this field. However, miRNA-based diagnosis and treatment in clinical practice face many challenges: the heterogeneity of the HDP phenotype must be taken into account, as well as the limitations of miRNA measurement and sampling methods and the need to reliably validate specific, accurate biomarkers in different patient groups, and the field remains heavily focused on descriptive expression profiling and individual mechanistic characterization, with a near-complete lack of large-scale prospective clinical trials validating biomarker performance or testing targeted miRNA-based therapeutic strategies.

Thus, future research should focus on elucidating the functional roles of specific miRNAs, shifting focus from individual molecule characterization to global ncRNA network analysis, prioritizing interaction between miRNAs, lncRNAs, circRNAs, and other regulatory molecules; improving diagnostic technologies; and confirming their clinical applicability. These advances hold the promise of improving the diagnostic accuracy, prognostic assessment, and therapeutic efficacy of HDP, ultimately promoting the health of mothers and neonates.

## 4 CircRNAs in hypertensive disorders of pregnancy

Following the extensive investigation of lncRNAs and miRNAs, circRNAs have emerged as another critical class of ncRNAs with unique structural and functional properties in human diseases. CircRNAs are a class of non-coding ribonucleic acids; their most distinctive feature is single-stranded molecules whose 3′- and 5′-ends are covalently linked to form a ring structure. It mostly consists of exons, introns, and noncoding intergenic regions, which are formed from mRNA precursors (pre-mRNA) by alternative splicing (Salzman et al., 2012; Memczak et al., 2013). Following miRNAs and lncRNAs, circRNAs constitute a new order within the ncRNA family and possess distinct properties, such as molecular diversity, evolutionary conservation, structural robustness, and cell-type-specific expression patterns (Memczak et al., 2013; Jeck et al., 2013). With the discovery and reporting of the growing family of circRNAs, their increasingly important biological roles are gradually becoming clearer: they can function as natural miRNA sponges to adsorb and regulate miRNA activity, as well as to regulate gene transcription through binding to transcriptional regulatory elements or interacting with proteins (Zhou W. Y. et al., 2020).

Over the past decade, many studies have focused on disorders of circRNAs associated with HDP in their pathophysiology, highlighting their role in pathogenesis and, in turn, expanding research in this field. Synthetic analysis of the field reveals a unifying regulatory paradigm: nearly all HDP-associated circRNAs function as ceRNAs to sponge target miRNAs, disrupting downstream effector gene expression and coordinately impairing core trophoblast functions, including proliferation, invasion, migration, apoptosis, epithelial-mesenchymal transition (EMT), and angiogenic signaling. This review provides a summary of current scientific knowledge on abnormally expressed circRNAs in patients suffering from HDP, assesses their functional role in disease progression, and explores their potential as new therapeutic targets. By proposing key mechanisms in the regulatory network of circRNAs, we attempt to propose new diagnostic and therapeutic strategies for the treatment of HDP.

### 4.1 The role of CircRNAs in modulating trophoblast cell functions

Given the central role of trophoblast dysfunction in the pathogenesis of HDP, understanding how circRNAs regulate trophoblast biology has become a major focus of recent research. In recent years, efforts have been made to explore the role of circRNAs in the mechanism of tumorigenesis. Due to the similarity between the biological behaviors of trophoblast cells and cancer cells, there was growing interest in circRNA-mediated trophoblast dysfunction during HDP development. Most dysregulated circRNAs in HDP uniformly disrupt core trophoblast biological processes via ceRNA-mediated miRNA sequestration, with expression profiles split into two pathological categories—upregulated circRNAs and downregulated circRNAs. (Tables 5, 6).

**TABLE 5:** The Upregulation and Roles of circRNAs in Trophoblast Cells during HDP.

CircRNAs	Expression levels	Mechanism of action	Detail mechanism	Function of the cells				Numbers of sample		Ref.
				Proliferation	Invasion	Migration	Apoptosis	CON	HDP	
circ_0011460	Up	Sponging miR-454–3p	circ_0011460→miR-454–3p↓→THBS2↑	inhibited	inhibited	inhibited	—	26	26	Ye et al. (2021)
circ_0026552	Up	Sponging miR-331–3p	circ_0026552→miR-331–3p↓→TGF-βR1↑	inhibited	inhibited	inhibited	—	30	30	Shan and Hou (2021)
circ_0037078	Up	Sponging miR-223–3p	circ_0037078→miR-223–3p↓→PI3K/AKT↓	inhibited	inhibited	inhibited	—	30	30	Tang et al. (2021)
circ_0029698	Up	Sponging miR-144	circ_0029698→miR-144↓→GRHL2↑	inhibited	inhibited	inhibited	—	15	26	Zhou B. et al. (2020)
circ_0111277	Up	Sponging miR-494	circ_0111277→miR-494↓→HTRA1↑→Notch-1↓	—	inhibited	inhibited	—	25	25	Ou Y. et al. (2020)
circ_0015382	Up	Sponging miR-149–5p	circ_0015382→miR-149–5p↓→TFPI2↑	inhibited	inhibited	inhibited	promoted	35	35	Hu et al. (2021)
circ_0002348	Up	Sponging miR-126–3p	circ_0002348→miR-126–3p↓→BAK1↑	inhibited	—	—	promoted	42	49	Zhou J. et al. (2023)
circ_0001438	Up	Sponging miR-942	circ_0001438→miR-942↓→NLRP3↑	inhibited	inhibited	inhibited	promoted	30	30	Li et al. (2021)
circ_0111277	Up	Sponging miR-424–5p	circ_0111277→miR-424–5p↓→NFAT5↑	—	inhibited	inhibited	—	30	30	Li and Li (2022)
circ_0005714	Up	Targeting sFLT1	circ_0005714→sFLT1↑	—	inhibited	—	—	32	32	Zhang et al. (2020b)

*THBS2,* Thrombospondin-2; *TGF-βR1,* Transforming growth factor-β receptor-1; *PI3K/AKT,* Phosphatidylinositol 3-kinase/protein kinase B; *GRHL2,* Grainy head-like 2; *HTRA1,* High-temperature requirement-A, serine peptidase 1; *Notch-1,* Notch receptor-1; *TFPI2,* Tissue factor pathway inhibitor 2; *NLRP3,* NOD-like receptor pyrin domain-containing protein 3; *NFAT5,* Nuclear factor of activated T-cell 5; *sFLT1,* Soluble fms-like tyrosine kinase 1.

**TABLE 6:** The Downregulation and Roles of circRNAs in Trophoblast Cells during HDP.

CircRNAs	Expression levels	Mechanism of action	Detail mechanism	Function of the cells				Numbers of sample		Ref.
				Proliferation	Invasion	Migration	Apoptosis	CON	HDP	
circ_0088227	Down	Sponging miR-384	circ_0088227→miR-384↑→STAT3↓	inhibited	inhibited	—	—	43	40	Zhou et al. (2019)
circ_0003496	Down	Sponging miR-1244	circ_0003496→miR-1244↑→FOXM1↓	inhibited	—	inhibited	—	25	25	Qi et al. (2021)
circ_0000284	Down	—	—	inhibited	inhibited	inhibited	ns	43	70	Zhang et al. (2019)
circCRAMP1L	Down	Targeting MSP	circCRAMP1L→MSP↑→RON↓	inhibited	inhibited	—	—	64	64	Conti et al. (2013)
circ_0036877	Down	Sponging miR-34a-5p	circ_0036877→miR-34a-5p↑→TFAP2A↓	inhibited	inhibited	inhibited	—	13	19	Zhang and Guo (2022)
circ_0001861	Down	Sponging miR-296–5p	circ_0001861→miR-296–5p↑→FOXP1↓	inhibited	inhibited	inhibited	—	39	52	Liu et al. (2024)

*STAT3,* Signal transducer and activator of transcription 3; *FOXM1,* Forkhead box protein M1; *MSP,* macrophage stimulating protein; *RON,* Recepteur d’origine nantais, (Receptor for macrophage stimulating protein); *TFAP2A,* targeting transcription factor AP-2α; *FOXP1,* Forkhead box protein 1; *ns,* no significance.

A hallmark of HDP pathogenesis involves impaired trophoblast function, characterized by aberrant EMT, reduced migratory capacity, and diminished invasiveness. The vast majority of HDP-associated upregulated circRNAs act as “pathological inhibitors” of trophoblast function. A representative example is the circ_0011460 (circAK2)/miR-454–3p/THBS2 (Thrombospondin-2) axis, identified as a promising regulatory pathway in PE: circ_0011460 is significantly overexpressed in HDP placental trophoblasts, where it competitively binds miR-454–3p and abrogates its inhibitory effect on THBS2, a multifunctional glycoprotein ubiquitously expressed across tissues that critically regulates cellular adhesion, proliferation, and motility (Ye et al., 2021). This series of events promotes the development of HDP, which directly suppresses trophoblast proliferation, migration, and invasion, driving the shallow placental invasion (Ye et al., 2021). In addition to this main pathway, several independent studies have confirmed the existence of parallel circRNA pathways, all of which ultimately lead to trophoblast dysfunction: circ_0026552, circ_0037078 (circLRRK1), circ_0029698 (circZDHHC20), and circ_0111277 exert similar inhibitory effects via miRNA-sponging mechanisms in different pathways, with their final targets including TGFβ-R1, PI3K/AKT, GRHL2, and Notch-1 signaling proteins (Shan and Hou, 2021; Tang et al., 2021; Zhou B. et al., 2020; Ou Y. et al., 2020). Furthermore, evidence suggests that circRNA regulation is involved in trophoblast cell apoptosis. The regulatory networks of circ_0015382/miR-149–5p/TFPI2 and circ_0002348/miR-126–3p/BAK1 have been linked to enhanced apoptotic signaling in trophoblast cells, highlighting the multiple roles of circRNA in HDP (Hu et al., 2021; Zhou J. et al., 2023). Furthermore, some circRNAs are highly expressed, directly promoting the apoptosis of trophoblast cells and stimulating the inflammatory response. For example, circ_0001438 contributes to inflammation and apoptosis via the miR-942/NLRP3 axis, extending the pathogenic consequences of circRNA dysfunction beyond mere cell motility (Li et al., 2021) (Table 5).

Conversely, a growing panel of circRNAs are consistently downregulated in PE placental and plasma samples, losing their physiological ability to sustain normal trophoblast function and exacerbating disease progression. These downregulated circRNAs typically act as “endogenous miRNA inhibitors.” Key representative examples include circ_0088227 (circPAPPA), circ_0003496 (circUBAP2), circ_0036877 (circFURIN), circ_0001861, and circCRAMP1L, all of which are depleted in PE tissues and/or plasma and promote trophoblast dysfunction via miRNA-dependent downregulation of STAT3, FOXM1, TFAP2A, FOXP1, and the MSP/RON signaling axis, respectively (Zhou et al., 2019; Qi et al., 2021; Zhang and Guo, 2022; Liu et al., 2024; Zhang et al., 2020a). Similarly, circ_0000284 (circHIPK3) downregulation correlates with severe PE and impaired trophoblast migration, invasion, and proliferation (Zhang et al., 2019). Collectively, these findings confirm that circRNA expression imbalance—either gain or loss of function—disrupts trophoblast homeostasis, with all regulatory axes unified by the ceRNA mechanism. All downregulated circRNA details are consolidated in Table 6.

As mentioned previously, dysfunction of angiogenesis is a significant cause of the development of HDP, a critical complementary pathway in HDP pathogenesis (Zhang et al., 2010; Conti et al., 2013). Research displayed that circ_0111277 and circ_0005714 (circSFXN1) are overexpressed in PE placentas, blocking trophoblast invasion and inhibiting endothelial cell angiogenesis via distinct downstream pathways, directly contributing to placental hypoperfusion (Li and Li, 2022; Zhang et al., 2020b). This dual regulatory role further solidifies circRNAs as central hubs in HDP pathogenesis, linking trophoblast cellular dysfunction to vascular defects.

### 4.2 CircRNA modulates epithelial-mesenchymal transition in trophoblast cells

Among the various functions of trophoblast cells regulated by circRNAs, epithelial-mesenchymal transition (EMT) stands out as a process of particular importance for early placental development and the pathogenesis of HDP. In the early stages of pregnancy, the vigorous invasion of extravillous trophoblasts (EVTs) is of the utmost importance for the development of the placental morphogenesis and for ensuring adequate blood for the fetus. Epithelial-mesenchymal transition (EMT) is a key process whereby epithelial cells are transformed into motile mesenchymal cells, which is beneficial for placental development (Lamouille et al., 2014). It is well established that aberrant EMT in trophoblast cells is associated with the progression of HDP (Dha et al., 2021). This section discusses recent studies on how circRNAs regulate EMT and affect the progression and severity of HDP.

As previously mentioned, new evidence shows that certain circRNAs are specifically involved in the development of trophoblastic cells. Not only do they regulate key functions, such as proliferation, migration, invasion, and apoptosis, but they also induce significant changes in the EMT process (Hu et al., 2021; Liu et al., 2024). A consistent and robust cross-study mechanistic theme emerges from synthetic analysis of existing literature: the vast majority of EMT-related circRNAs are significantly upregulated in PE placental tissues, and all act as potent endogenous suppressors of trophoblast EMT through the classic ceRNA mechanism, specifically by competitively sponging miRNAs that normally promote mesenchymal transition and trophoblast invasive function. Multiple independent studies have successively identified a panel of distinct upregulated circRNAs with consistent EMT-inhibitory effects, including circ_0006772 (circTNRC18), circ_0111277, circ_0000566 (circVRK1), circ_0008726, and circ_0001326; although each of these circRNAs binds to different target miRNAs and regulates unique downstream mRNA molecules, their functional outcomes converge uniformly on blocking trophoblast EMT and reducing cell invasiveness (Shen et al., 2019; Zhou M. et al., 2023; Li et al., 2022; Shu C. et al., 2022; Gao et al., 2023). The core downstream effector molecules modulated by these axes mainly include GRHL2, PTEN/Akt, RYBP, and HTRA1, all of which are key regulatory genes that dominate the process of EMT (Shen et al., 2019; Zhou M. et al., 2023; Li et al., 2022; Shu C. et al., 2022; Gao et al., 2023).

In contrast to the upregulated circRNAs that inhibit the EMT, there is a small group of circRNAs that are reduced in HDP placental samples and are equally functionally highly significant. The downregulation of those circRNAs disrupts the normal EMT of trophoblast cells by inhibiting target genes. Representative members of this group include circ_0007121 and circ_0032962, which exert their regulatory effects via two distinct molecular axes: circ_0007121 regulates the miR-182–5p/PGF axis to inhibit EMT in HTR-8/SVneo trophoblast cells, while circ_0032962 impairs EMT via the miR-326/PBX3 axis, both of which ultimately contribute to the pathological EMT in HDP (Gai et al., 2020; Mao and Zou, 2021). Notably, several HDP-associated circRNAs do not regulate EMT in isolation but exert dual or multifaceted regulatory effects on trophoblast biology; for example, circ_0001861 not only directly suppresses trophoblast EMT progression but also impairs basal trophoblast proliferation, migration, and invasive capacity simultaneously, which further amplifies the pathological damage to placental development. This dual regulatory pattern fully highlights the integrated and multi-target nature of circRNA-mediated regulation in HDP pathogenesis, rather than acting through single isolated molecular events (Liu et al., 2024).

**FIGURE 2:**
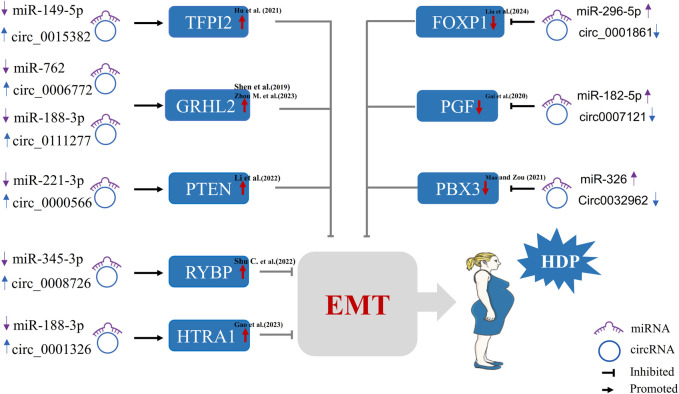
CircRNAs modulate epithelial-mesenchymal transition (EMT) in trophoblast cells during the HDP. (*TFPI2,* Tissue factor pathway inhibitor 2; *GRHL2,* Grainy head-like 2; *PTEN,* Phosphatase and tensin homolog deleted on chromosome 10; *RYBP,* RING1 and YY1 binding protein; *HTRA1,* High-temperature requirement-A serine peptidase 1; *FOXP1,* Forkhead box protein 1; *PGF,* Placental growth factor; *PBX3,* Pre-B-cell leukemia homeobox 3).

This complex circRNA-miRNA-mRNA regulatory network serves as a unified and powerful mechanism underlying the pathological changes observed in HDP. Across all studies, no conflicting mechanistic findings have been reported regarding circRNA-mediated EMT regulation: every dysregulated circRNA in HDP directly or indirectly inhibits trophoblast EMT, regardless of the specific miRNA-mRNA axis involved. This unidirectional functional convergence simplifies the core message and underscores the critical role of circRNAs in HDP progression, as Figure 2 showed.

### 4.3 Possibility: circRNAs as potential targets for HDP

The role played by circRNA in the pathogenesis of diseases associated with HDP was the main focus of current investigation. Some studies have identified specific circRNA expression patterns that govern placental tissue in patients with PE. Given their stability and high detectability in biological fluids such as blood, urine, and exosomes, circRNAs have great potential as a diagnostic marker for detecting abnormalities associated with HDP. Zhang et al. demonstrated that combining plasma protein markers and circRNAs improves the accuracy of PE prediction; however, these findings need to be confirmed in studies involving various clinical cohorts (Zhang et al., 2016). Using bioinformatic analysis, Jiang et al. identified circ_0004904 and circ_0001855 in blood samples that may interact with pregnancy-associated plasma protein-A and contribute to the development of PE. This association with pregnancy-associated plasma protein A causes them to evolve as diagnostic markers of PE (Jiang et al., 2018). All of this demonstrates the practical potential of circRNAs to evolve as biomarkers of HDP.

In this review, we comprehensively review the most important circRNAs whose expression changes in HDP and explain their mechanisms of action. The final objective is to identify new molecular targets for prevention, diagnosis, and therapeutic intervention. Nevertheless, no circRNA-based biomarkers have yet entered clinical trials or routine clinical use, and mechanistic research remains focused on individual circRNA-miRNA-mRNA axes, with no comprehensive studies exploring global circRNA regulatory networks or interaction with other ncRNA species. Highlighting the urgent need for further validation of their accuracy, sensitivity, and regulatory networks in future studies.

## 5 Integrative mechanistic insights: the role of ncRNA regulatory networks in HDP pathogenesis

Given the vast variety and large number of ncRNAs, current ncRNA research remains fragmented by individual RNA class characterization, with little cross-class integration to identify core regulatory hubs driving disease pathogenesis, making it difficult at this time to compile a comprehensive interaction network diagram.

However, upon reviewing the existing literature, we found synthetic analysis reveals a unifying regulatory cascade: lncRNAs and circRNAs act as upstream master regulators, sponging downstream effector miRNAs to modulate target gene expression, and all three ncRNA classes converge on the core pathological pathways of HDP—impaired trophoblast invasion, defective spiral artery remodeling, aberrant EMT, and excessive placental oxidative stress. Notably, not all ncRNAs operate within a ceRNA-mediated interaction axis. A substantial body of existing research has documented that most lncRNAs and miRNAs contribute to the initiation and progression of HDP either through direct regulatory effects or by targeting their respective downstream effector genes. This observation implies two possibilities: either these ncRNAs directly govern core cellular functions relevant to placental development and vascular homeostasis in an independent manner, or the current evidence supporting ceRNA-mediated interactions—or confirming the upstream regulatory cascades that link different miRNAs—remains limited. This suggests that critical mechanistic gaps and missing regulatory links within the ncRNA functional network need to be fully elucidated.

**FIGURE 3:**
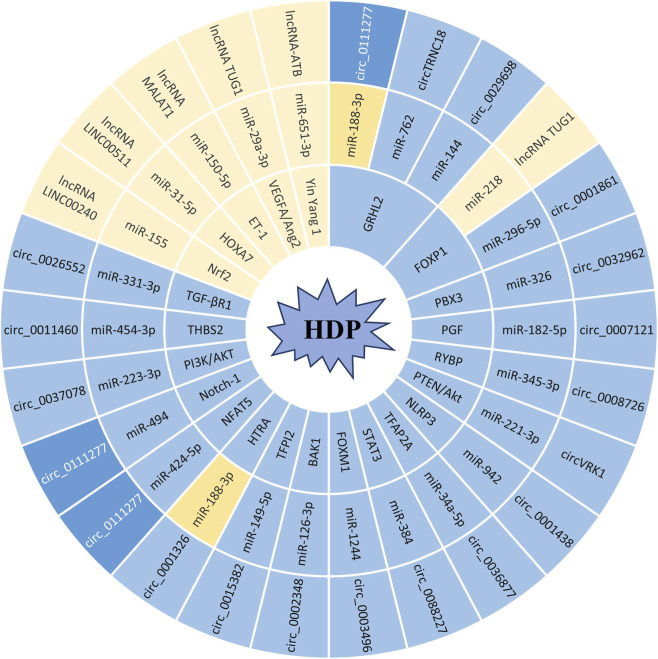
The Role of ncRNA Regulatory Networks in HDP Pathogenesis.

To address this gap, we collated and integrated validated ncRNA interaction axes from published studies and constructed a ncRNA regulatory interaction network to visualize cross-class interaction and core regulatory hubs. As clearly illustrated in Figure 3, several pivotal molecules with exceptional functional and translational relevance stand out: circ 0111277 exerts pleiotropic regulatory effects through multiple parallel signaling pathways, underscoring its immense potential as a robust non-invasive diagnostic biomarker for HDP (Ou Y. et al., 2020; Li and Li, 2022; Zhou M. et al., 2023). The miR-188–3p functions as a core downstream effector molecule targeted by a panel of lncRNAs and circRNAs, highlighting its irreplaceable mediating role in the global ceRNA regulatory cascade (Zhou M. et al., 2023; Gao et al., 2023). Additionally, GRHL2 and FOXP1 serve as a convergent downstream target for multiple dysregulated lncRNAs and circRNAs, indicating their status as a promising and actionable therapeutic candidate for HDP-targeted intervention (Xu et al., 2022; Zhou B. et al., 2020; Liu et al., 2024; Shen et al., 2019; Zhou M. et al., 2023). Regarding disease progression, ncRNAs dysregulated in early gestation, localized to trophoblasts, and directly triggering vascular remodeling defects (e.g., lncRNA MALAT1, miR-210–5p, and circ_0088227) could be primary drivers of HDP (Li et al., 2020; Awamleh and Han, 2020; Zhou et al., 2019). Those achievements serve as a reference for the integration of global ncRNA networks in HDP research.

## 6 Conclusion

HDP are serious conditions that affect the health of both the mother and the child during pregnancy, as well as their long-term outcomes. After reviewing the roles of lncRNAs, miRNAs, and circRNAs in HDP, it is clear that ncRNAs have reshaped our understanding of the molecular mechanisms underlying these complex pregnancy disorders. However, both accurately predicting the onset risk and developing treatments for HDP have become particularly difficult due to the heterogeneity of its clinical presentation and the complexity of its pathogenesis. Currently, the only available solution in the most severe cases is termination of the pregnancy, which emphasizes the urgent necessity of novel therapeutic strategies and early diagnostic tools. In the last 10 years, significant advancements have been made in understanding the role of ncRNAs in the pathophysiology of HDP, such as lncRNAs, miRNAs, and circRNAs. These ncRNAs have been shown to regulate the functions of trophoblastic cells, spiral artery remodeling, vascular endothelial cell dynamics, and EMT. These processes are necessary for the proper development and function of the placenta; Their disruption leads to the key features of HDP, such as structural placental abnormalities and systemic vascular dysfunction.

The ncRNAs have emerged as a promising biomarker and therapeutic target for the treatment of HDP, opening up new opportunities for precision medicine. Longitudinal studies conducted that before the onset of clinical symptoms, abnormal ncRNA profiles can be detected in maternal blood from early pregnancy, and these profiles differ between normotensive pregnancies and HDP (Zhou S. et al., 2023; Moufarrej et al., 2022). This research emphasizes the potential of circulating ncRNA as a non-invasive prognostic marker in early pregnancy. However, most research has focused on lncRNAs, miRNAs, and circRNAs, other types of ncRNAs, such as transfer RNAs, ribosomal RNAs, small nuclear RNAs, small interfering RNAs, and Piwi-interacting RNAs, have not been extensively studied. Investigation on the role of these less studied ncRNAs in HDP will not only provide a deeper understanding of the underlying mechanisms but also expand the range of potential biomarkers and therapeutic targets.

Although the research on ncRNAs as biomarkers has a promising future, there still remain a number of challenges that need to be resolved. Current methods for detecting circulating ncRNA in blood are often time-consuming and expensive, which hinders their widespread application. Furthermore, standardisation of detection protocols; large-scale prospective cohort validation; the impact of differences in sample collection, storage, RNA extraction methods, and sequencing platforms on the accuracy of research findings; and potential instability and off-target effects in ncRNA drug development, as well as barriers to clinical application, are excellent challenges that need to be faced. To achieve the clinical translation of ncRNA in HDP, these technical and methodological limitations need to be addressed in future research.

This review consolidates the significant advances in elucidating the role of lncRNAs, miRNAs, and circRNAs in HDP pathogenesis and also identified critical knowledge gaps and challenges. Once these challenges have been overcome, it will be possible to accelerate the translation of ncRNA research into clinical trials and treatments, thus improving health outcomes for mothers and infants affected by HDP.
